# Editorial: Molecular Mechanisms of Voltage-Gating in Ion Channels

**DOI:** 10.3389/fphar.2021.768153

**Published:** 2021-09-15

**Authors:** Gildas Loussouarn, Mounir Tarek

**Affiliations:** ^1^ L'Institut Du Thorax, Inserm UMR 1087/CNRS UMR 6291, Nantes, France; ^2^ Université de Lorraine, LPCT, CNRS UMR 7019, Nancy, France

**Keywords:** voltage-gated ion channel, patch - clamp technique, toxins, cryo-EM, VSD gate coupling

Voltage-gated ion channels are transmembrane proteins conducting ions according to the electrochemical gradient, when opened by voltage. Hence, in these channels, at least one of the channel gates regulating the ion flux is controlled by the transmembrane potential. They are frequently ion specific and therefore selectively permeable to sodium (Na_V_ channels), potassium (K_V_ channels), calcium (Ca_V_ channels) or chloride (CLC channels) ions. Depending on the channels, opening of the activation gate is triggered by membrane depolarization (e.g. K_V_, Na_V_ and Ca_V_ channels) or hyperpolarization (HCN channels for instance). In addition, in many voltage-gated channels, a so-called inactivation gate is also present. Compared to the activation gate, the latter is, when voltage-dependent, oppositely coupled to the potential: In K_V_, Na_V_ and Ca_V_ channels, upon membrane depolarization, the inactivation gate closes whereas the activation gate opens.

Various voltage-dependent channels have been identified, depending on the excitable cell types in which they are expressed and their physiological role. They are characterized by their conductance, ion selectivity, pharmacology and voltage-sensitivity. These properties are mainly dictated by the amino-acid sequence and structure of the pore forming subunit(s), the presence of accessory subunit(s), the membrane composition and the intra- and extracellular ions concentrations. Many mutations have been identified in these channels, impacting their functions and provoking diseases named channelopathies.

In 2012, we hosted a Research Topic on the Molecular Mechanisms of Voltage Dependency (Loussouarn and Tarek, 2012), bringing together scientists to collaborate and showcase the latest developments in the field. Since this Frontiers Research Topic, the development of new approaches, such as the use of cryo-electron microscopy (cryo-EM) at the atomic scale and the original approach of split channels, to name a few, has led to a more precise understanding of the mechanisms of voltage-gating, their targeting by toxins, and also their physio-pathological implications. Given the wealth of recent electrophysiological, biochemical, optical, and structural data regarding ion channel voltage-dependence, we felt there was clearly a need for putting together a new Research Topic that would include up to date Reviews and Original Research describing molecular details of the functioning of these complex voltage-gated channels.

The review of Brewer et al. underlines how ion channel structures and models reveal critical differences in the atomic details of KCNQ1, hERG, and Na_V_1.5 structures associated with their distinct voltage-gating and implication in Long QT syndrome, and also their pharmacological profiles. Such structural data may help defining the pathogenicity of the hundreds of variants in absence of functional data. It also mentions an important point: Molecular Dynamics represents a useful tool in refining cryo-EM structures, which are often of lower resolution in the periphery of protein core structures.

Among the critical differences recapitulated by Brewer et al., cryo-EM was instrumental to reveal the presence of a variation in the overall organization of voltage-gated channels, which is the presence of a non-domain-swapped *vs.* a domain-swapped structure. The non-domain-swapped structure (in which the voltage sensor of subunit A leans against the pore-forming helices of subunit A, B against B, etc.) first identified in K_V_10.1 channel and confirmed in hERG/K_V_11.1 has a major molecular impact: it prevents the role of the S4-S5 linker as a mechanical lever, and may explain why K_V_10.1 and K_V_11.1 channels, when artificially split at the level this S4-S5 linker, are still voltage-gated (Barros et al.). These observations are greatly compatible with the ligand-receptor suggested by our work on K_V_10.2 and K_V_11.2 (Malak et al., 2019; Malak et al., 2017). It remains puzzling then that such a ligand-receptor model also applies to the domain-swapped K_V_7.1 channel (Choveau et al., 2011), suggesting that key elements are still missing to understand the molecular mechanism of voltage gating.

Another article of our Research Topic also underlines the role of Structural Biology data, enriched by Molecular Dynamics, to confirm and refine the succession of electrostatic interactions of S4 residues with S1 to S3 countercharges during voltage-gated activation, and the role of voltage sensor domain (VSD) hydration in ion channel functioning (Groome and Bayless-Edwards). Importantly, some mutations of countercharges present in VSD are implicated in neural, cardiac and skeletal muscle disorders. For example, in hERG, mutation of these countercharges accelerates deactivation, slow deactivation being crucial for proper repolarization of the ventricular action potential (Shi et al.). VSD also seems to play a role in hysteresis of channel gating. Hysteresis is a property of channel gating making it dependent on its “history,” resulting, for hERG, in a shifted voltage-dependence of deactivation towards hyperpolarized potentials as compared to the voltage-dependence of activation. Structural determinants of this property have been reviewed for hERG in Shi et al., suggesting a contribution of both the S4-S5 linker and the VSD to hERG gating hysteresis. Hysteresis has been more generally and very didactically reviewed in Villalba-Galea and Chiem, including physiological implications. For instance, hysteresis prevents HCN activation during the repolarization phase of the sinoatrial node action potential.

Regarding voltage-gated channel modulators targeting hydrophobic binding sites, the review of Van Theemsche et al. compiled the residues implicated in K_V_ channel modulation by various lipophilic molecules. Mapping them on available structural data, they suggest three lipophilic drugs/toxin binding sites, a first step toward the classification of hydrophobic binding sites in the large and complex family of K_V_ channels.

Finally, we are glad that our Research Topic motivated the submission of original articles. Kudaibergenova et al. compared the molecular mechanisms of ivabradine and dofetilide block of hERG channel. Li et al. correlated the clinical response to mexiletine of LQT3 patients carrying various mutation on *SCN5A* (coding for Na_V_1.5), to the extent of inhibition of the late Na current by mexiletine, in cells expressing these mutated Na_V_1.5 channels. Chang et al., described the off-target effect of remdesivir, an antiviral drug that was recently tested in SARS-CoV2 infected patients, on voltage-gated potassium currents present in pituitary tumor (GH3) and T lymphoblast (Jurkat) cells. Inwardly rectifying channels are not voltage-gated channels strictly speaking, but pore block by polyamines, the molecular mechanism of rectification in these channels, is a voltage-dependent process. For that reason and because tools are the same to study inwardly rectifying channels and voltage-gated channels, we also included a study that used Molecular Dynamics to get further insights on the mechanism of voltage dependent block by the polyamine putrescine (Chen et al.).
